# [*O*-*methyl*-^11^C]*N*-(4-(4-(3-Chloro-2-methoxyphenyl)-piperazin-1-yl)butyl)-1*H*-indole-2-carboxamide ([^11^C]BAK4-51) Is an Efflux Transporter Substrate and Ineffective for PET Imaging of Brain D_3_ Receptors in Rodents and Monkey

**DOI:** 10.3390/molecules23112737

**Published:** 2018-10-23

**Authors:** Jeih-San Liow, Cheryl L. Morse, Shuiyu Lu, Michael Frankland, George L. Tye, Sami S. Zoghbi, Robert L. Gladding, Anver B. Shaik, Robert B. Innis, Amy H. Newman, Victor W. Pike

**Affiliations:** 1 Molecular Imaging Branch, National Institute of Mental Health, National Institutes of Health, Room B3C346, 10 Center Drive, Bethesda, MD 20892, USA; liowj@mail.nih.gov (J.-S.L.); morsec@mail.nih.gov (C.L.M.); michael.frankland@nih.gov (M.F.); george.tye@gmail.com (G.L.T.); zoghbis@mail.nih.gov (S.S.Z.); gladdingr@mail.nih.gov (R.L.G.); innisr@mail.nih.gov (R.B.I.); pikev@mail.nih.gov (V.W.P.); 2 Medicinal Chemistry Section, Molecular Targets and Medications Discovery Branch, National Institute on Drug Abuse, National Institutes of Health, 333 Cassell Drive, Baltimore, MD 21224, USA; aner.shaik@nih.gov (A.B.S.); anewman@intra.nida.nih.gov (A.H.N.)

**Keywords:** dopamine D_3_ receptors, antagonist, radiolabeling, PET, efflux transporter substrate

## Abstract

Selective high-affinity antagonists for the dopamine D_3_ receptor (D_3_R) are sought for treating substance use disorders. Positron emission tomography (PET) with an effective D_3_R radioligand could be a useful tool for the development of such therapeutics by elucidating pharmacological specificity and target engagement in vivo. Currently, a D_3_R-selective radioligand does not exist. The D_3_R ligand, *N*-(4-(4-(3-chloro-2-methoxyphenyl)piperazin-1-yl)butyl)-1*H*-indole-2-carboxamide (BAK4-51, **1**), has attractive properties for PET radioligand development, including full antagonist activity, very high D_3_R affinity, D_3_R selectivity, and moderate lipophilicity. We labeled **1** with the positron-emitter carbon-11 (*t*_1/2_ = 20.4 min) in the methoxy group for evaluation as a radioligand in animals with PET. However, [^11^C]**1** was found to be an avid substrate for brain efflux transporters and lacked D_3_R-specific signal in rodent and monkey brain in vivo.

## 1. Introduction

The dopamine D_3_ receptor (D_3_R), a member of the dopamine D_2_-like receptor family, is highly localized in neurocircuits that play an important role in emotional and cognitive functions [1,2,3,4,5]. Aberrant D_3_R signaling has been linked to multiple neurological and psychiatric conditions, such as Parkinson’s disease [6], schizophrenia [6], behavioral sensitization [7], depression [8], and substance use disorders (SUD) [9,10,11].

**Chart 1:**
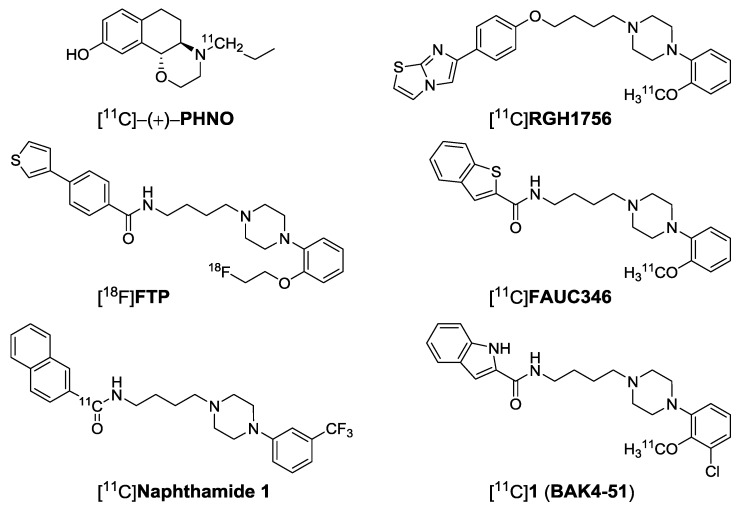
Structures of some former candidate D_3_R PET radioligands and [^11^C]**1**.

Positron emission tomography (PET) can provide quantitative information on the distribution of many brain proteins of biomedical interest (e.g., neuroreceptors) in healthy and diseased states of living subjects through the measurement of selective radioligand distribution. PET can also assist in the development and evaluation of potential therapeutics by, for example, providing information on target engagement under prescribed trial dosing regimens [12,13,14,15,16]. The availability of effective radioligands is key to exploiting the full potential utility of PET in drug development. In an in-depth review [17], Mach and Luedtke recently discussed the gap between the need for D_3_R-specific PET radioligands and the challenges encountered in their development. An enormous medicinal chemistry effort has led to the discovery of many highly selective D_3_R ligands but no effective PET radioligand for D_3_R has emerged. Two key hurdles to PET radioligand development are (a) how to design a ligand that is able to differentiate between D_2_R and D_3_R, which share ~80% homology within ligand binding domains [18], and (b) how to overcome the coexistence of D_2_R and D_3_R in several brain regions, such as the D_3_R-rich striatum where the density of D_2_R exceeds [19] the density of D_3_R by ratios that are reported to be at least 2:1 [20,21,22], dependent on measurement methodology. A few high-affinity and selective D_3_R ligands have been evaluated as candidate D_3_R PET radioligands ([^11^C]PHNO [21,23]; [^11^C]RGH1756 [24]; [^18^F]FTP [25]; [^11^C]FAUC 346 [26] and [^11^C]Naphthamide 1 [27]) (Chart 1), but none has so far proved wholly effective [28]. Various factors, including insufficiently high D_3_R affinity or the possibility that D_3_R are highly occupied by endogenous dopamine in vivo [21], may be responsible for the lack of efficacy in these radioligands. [^11^C]PHNO has been widely used for PET studies of D_3_R in human subjects but suffers from contamination of the PET signal in many brain regions by a high proportion of signal from D_2_R [29]. This lack of selectivity exacerbates the difficulty of brain D_3_R quantification.

In our search for high-affinity dopamine D_3_R-selective antagonists for treating SUD, we have discovered several D_3_R-selective ligands that may be suitable for development as PET radioligands [30]. To further understand the molecular basis for pharmacological specificity towards the D_3_R and in particular, to provide a biomarker for receptor engagement in our efforts toward pharmacotherapeutic development, we chose one of the highest affinity (*h*D_3_R *K*_i_, 0.4 nM) and D_3_R-selective (*h*D_2_R *K*_i_, 53 nM) full antagonists in this series, namely *N*-(4-(4-(3-chloro-2-methoxyphenyl)piperazin-1-yl)butyl)-1*H*-indole-2-carboxamide (BAK4-51; **1**) [30] as a prospective PET radioligand. We report the synthesis of [^11^C]**1** (Chart 1) and its evaluation with PET in rodents and monkey.

## 2. Results and Discussion

### 2.1. Lead Compound Selection

Compound **1** was recently identified as a lead D_3_R-selective antagonist [30]. For this chemotype, the 4-phenylpiperazine moiety binds to the highly homologous D_2_R/D_3_R orthosteric binding site, whereas the indole-2-carboxamide terminus binds in a secondary binding pocket that differs topographically between D_2_R and D_3_R [31,32,33]. Incorporation of a 2-OCH_3_, 3-Cl-substituted phenyl ring and a 4-carbon linker resulted in a high affinity ligand at D_3_R (D_3_R *K*_i_ = 0.39 ± 0.04 nM, D_2_R *K*_i_ = 53.0 ± 6.2) with 136-fold selectivity for D_3_R over D_2_R [30]. Functional assay and off target binding assay data showed that **1** is a full antagonist (*IC*_50_ = 40 nM in the mitogenesis assay in *h*D_3_R transfected CHO cells) and exhibited relatively low affinities toward other neuroreceptors compared to D_3_R, as demonstrated by the *K*_i_ values for D_1_ (2290 ± 340 nM), D_4_ (300 ± 100 nM), 5-HT_1A_ (92.3 ± 8.0 nM), 5-HT_2A_ (19.7 ± 1.4 nM) and 5-HT_2C_ (51 ± 11 nM) receptors. cLog*D* for compound **1** is 2.19 and the CNS MPO score is >3, which are within the ranges deemed suitable for penetration of the blood-brain barrier (BBB) [34,35,36]. The 2-OCH_3_ substituent provides a suitable site for labeling with carbon-11.

### 2.2. Radiolabeling of [^11^C1]

**Figure 1:**
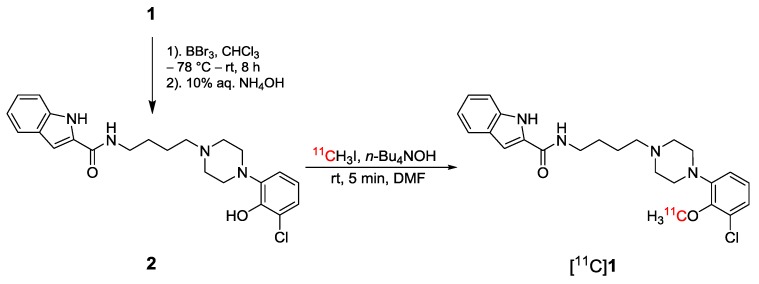
Radiosynthesis of [^11^C]**1** from its desmethyl precursor **2** and [^11^C]iodomethane. Precursor **2** was prepared from compound **1** by treatment with BBr_3_.

*O*-Demethylation of **1** [30] with BBr_3_ gave the phenol **2** in 65% yield for use as a precursor for radiolabeling. Treatment of **2** with [^11^C]iodomethane in the presence of a weak base, *n*-Bu_4_NOH, in DMF at room temperature for 5 min with the ‘loop method’ [37] readily gave [^11^C]**1** (Figure 1), which was purified with reversed phase HPLC and formulated for intravenous injection. The yield of formulated [^11^C]**1** was 8 ± 3% (*n* = 8) from cyclotron-produced [^11^C]carbon dioxide, the radiochemical purity greater than 99%, and the molar activity (*A*_m_) 251 ± 83 GBq/μmol (*n* = 8) at the end of synthesis and formulation (about 45 min from the start of radiosynthesis). The high *A*_m_ value shows that the starting desmethyl precursor (**2**) was obtained free of any significant amount of contamination with residual carrier (**1**). Reversed phase HPLC of the reaction mixture revealed two radioactive byproducts with shorter retention time (*t*_R_ ≤ 7 min) than [^11^C]**1** (*t*_R_ = 10–11 min), indicating possible ^11^C-methylation competition at the amide and/or indole nitrogens. However, since [^11^C]**1** was well separated from these byproducts and its desmethyl precursor **2** (*t*_R_ = 6 min), no further development of the separation was undertaken. [^11^C]**1** was highly stable in saline containing ethanol (10% *v*/*v*) for up to 2.5 h because HPLC analysis of the formulated radioligand solution showed that radiochemical purity decreased only slightly from 99% to 96%.

### 2.3. LogD_7.4_, Stability and Plasma Free Fraction for [^11^C]1

The log*D***_7.4_** of [^11^C]**1** at room temperature was found to be 3.33 ± 0.13 (*n* = 6), and somewhat higher than calculated. However, this value is still within the lipophilicity range that is regarded as desirable for PET radioligands targeting brain [38]. Human plasma free fraction (*f*_p_) was low (0.15 ± 0.01%; *n* = 3). Although a higher and more accurately measurable *f*_p_ value would be desirable for PET studies, low *f*_p_ does not strictly preclude PET radioligand utility, as shown with [^11^C]MePPEP (*f*_p_ = 0.05 ± 0.01%) [39]. [^11^C]**1** demonstrated good stability for at least 30 min at 37 °C in rat whole blood (100%), human (99.3%), and rat plasma (98.8%), and in rat brain (99.4%), monkey brain (100%), and human brain (99.8%) homogenates. The cellular uptake in rat whole blood in vitro at 37 °C was 55.2 ± 1.1% (*n* = 3). High cell uptake could be one of the many factors that counter the ability of a radioligand to readily enter brain. Ex vivo evaluation showed that in rat plasma there were at least five radiometabolites with much less lipophilicity than [^11^C]**1**, based on shorter reversed phase HPLC retention times. Under baseline conditions 76% of the radioactivity in the plasma at 30 min after administration of [^11^C]**1** was unchanged radioligand (Table 1). In some experiments tariquidar [40] was pre-administered at doses in the range 4 to 16 mg i.v. to inhibit the action of the efflux transporters P-glycoprotein (P-gp) and breast cancer resistance protein (BCRP) at the BBB [41,42]. This treatment had little effect on the radiometabolite profile in plasma as 69% of the radioactivity in the plasma at 30 min after administration of [^11^C]**1** was unchanged radioligand (Table 1). Nearly all radioactivity measured in brain tissues was unmetabolized [^11^C]**1** (Table 1).

### 2.4. [^11^C]1 Is a Brain Efflux Transporter Substrate in Rat, Mouse, and Monkey

Brain tissue radioactivity concentrations (SUV) in rats under baseline and tariquidar-treated (16 mg/kg, i.v.) conditions, measured ex vivo at 30 min after intravenous injection of [^11^C]**1**, are summarized in Table 2. Whole blood and plasma concentrations of [^11^C]**1** were similar in tariquidar-treated and untreated rats. Whole blood and plasma concentrations were the same before and after tariquidar treatment. In tariquidar-treated rats, the concentrations of [^11^C]**1** in rat brain tissue were markedly higher than in untreated rats by 2.4–2.6 fold, indicating a significant action of the efflux transporter, P-gp, on [^11^C]**1** brain uptake under baseline conditions. Ratios of [^11^C]**1** in cortex or striatum to cerebellum (1.1–1.2) were similar in tariquidar-treated and untreated rats, indicating little or no preference for [^11^C]**1** to bind to D_3_R-rich non-cerebellar regions.

**Figure 2:**
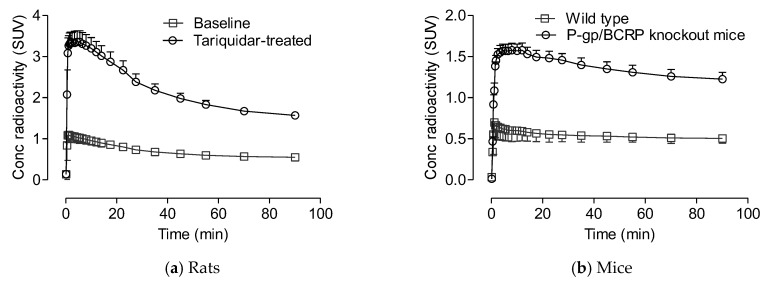
(**a**) Whole brain PET TACs for [^11^C]**1** in rats under baseline (*n* = 3) and tariquidar-treated (*n* = 3) condition, and in wild type (*n* = 2) and (**b**) P-gp/BCRP knock-out (*n* = 3) mice. Data are mean ± SD for *n* = 3 and mean ± range for *n* = 2. One-sided error bars represent standard deviation.

PET imaging of rats before and after tariquidar treatment (16 mg/kg, i.v.) provided similar findings to those obtained by ex vivo studies. Peak brain radioactivity concentration increased from 1.1 to 3.4 SUV (Figure 2a) after P-gp inhibition. The striatum to cerebellum radioactivity concentration ratio (≈1) was unchanged when P-gp was inhibited (time-activity curves not shown). Moreover, the relatively high uptake of [^11^C]**1** in cerebellum does not accord with the low density of D_3_R in cerebellum observed in vitro [20,21,22]. These results may indicate some off-target and/or high non-specific binding.

To further explore the action of efflux transporters on the brain uptake of [^11^C]**1**, and to test whether BCRP may act on [^11^C]**1**, PET experiments were performed in wild type and P-gp/BCRP double knock-out (KO) mice. Uptake of [^11^C]**1** in the brain was higher in the P-gp/BCRP KO mice than in wild type mice (peak uptake 1.6 vs. 0.7 SUV) (Figure 2b). These results clearly demonstrated that [^11^C]**1** is an avid brain efflux transporter substrate in rodents. Many formerly tested candidate radioligands for D_3_R have strong structural similarity to [^11^C]**1** (Chart 1) but it is unreported whether these radioligands, some of which show low brain uptake, are also substrates for brain efflux transporters.

**Figure 3:**
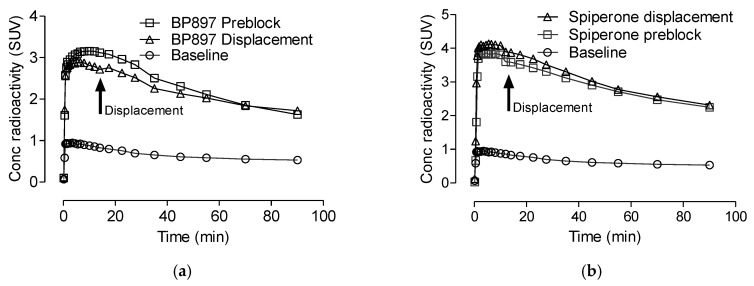
(**a**) PET TACs after administration of [^11^C]**1** to rats (i) at baseline (◯, *n* = 1), (ii) after pretreatment with tariquidar (16 mg/kg, (i.v.) plus BP897 (0.5 mg/kg, (i.v.) (□, *n* = 1), and (iii) after pretreatment with tariquidar (16 mg/kg, (i.v.), and then with BP897 (0.5 mg/kg, i.v) at 15 min after [^11^C]**1** (∆, *n* = 1). (**b**) PET TACs after administration of [^11^C]**1** to rats (i) at baseline (◯, *n* = 1), (ii) after pretreatment with tariquidar (16 mg/kg, (i.v.) plus spiperone (1.0 mg/kg, (i.v.) (□, *n* = 1), and iii) after pretreatment with tariquidar (16 mg/kg, (i.v.), and then with spiperone (1.5 mg/kg, (i.v) at 15 min after [^11^C]**1** (∆, *n* = 1).

To test whether any of the uptake of [^11^C]**1** in rat brain represented D_3_R-specific binding, experiments were performed with pharmacological challenge from another high-affinity selective D_3_R ligand, BP897 (D_3_R *K*_i_ = 0.92 nM) which has been used to displace [^11^C]PHNO in vivo [43]. Two rats were each treated with tariquidar (16 mg/kg, i.v.) 15 min before injection of [^11^C]**1**. One rat was also treated with BP897 (0.5 mg/kg, i.v.) at 10 min before radioligand injection, and the other with BP897 (0.5 mg/kg, i.v) at 15 min after radioligand injection. No major changes in the shapes of TACs were observed as a result of BP897 administration (Figure 3a).

The D_3_R subtype shares a substantial portion of amino acid sequence and tissue distribution with the D_2_R subtype but is much less abundant and has more restricted tissue localization than the D_2_R subtype [18,32]. We suspected that some of the rat brain radioactivity uptake might be bound to D_2_R. To test this possibility, pre-blocking and displacement experiments were also carried out using a selective D_2_-like receptor ligand, spiperone (D_2_R, *K*_i_ = 0.2 nM) [44,45,46]. Two rats were each treated with tariquidar (16 mg/kg, i.v.) at 15 min before injection of [^11^C]**1**. One was treated with spiperone (1.0 mg/kg, i.v.) at 10 min before radioligand injection and the other with spiperone (1.0 mg/kg, i.v.) at 15 min after radioligand injection. No obvious changes in the TACs were observed after administration of spiperone (Figure 3b). The striatum to cerebellum ratio changed slightly from 1.0 to 1.3 from 20 min to the end of scan sessions. However, these ratios were indistinguishable between baseline, preblocking, and displacement conditions. Therefore, we found no clear evidence for off-target binding of [^11^C]**1** to D_2_R in vivo.

**Figure 4:**
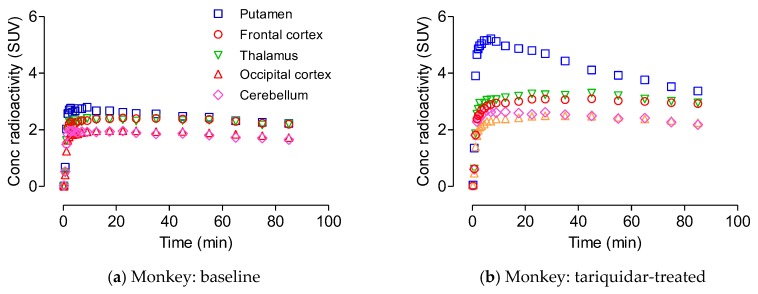
Time-activity curves of in monkey administered [^11^C]**1** under baseline (**a**) and tariquidar-pretreated conditions (**b**).

In monkey PET experiments, the peak whole brain radioactivity uptake after intravenous administration of [^11^C]**1** was moderate (2−3 SUV) and early at baseline. Peak uptakes of radioactivity in putamen, frontal cortex, thalamus and occipital cortex were somewhat higher than in cerebellum (Figure 4a). Many other brain regions not shown in Figure 4a gave TACs that resembled those for the shown non-cerebellar regions. Peak radioactivity concentrations declined at a very slow rate from all brain regions over the 90-min scan period. The ratio of radioactivity in putamen to that in cerebellum was 1.4 at both 9 and 35 min after injection, thereby indicating low or absent D_3_R specific binding.

**Figure 5:**
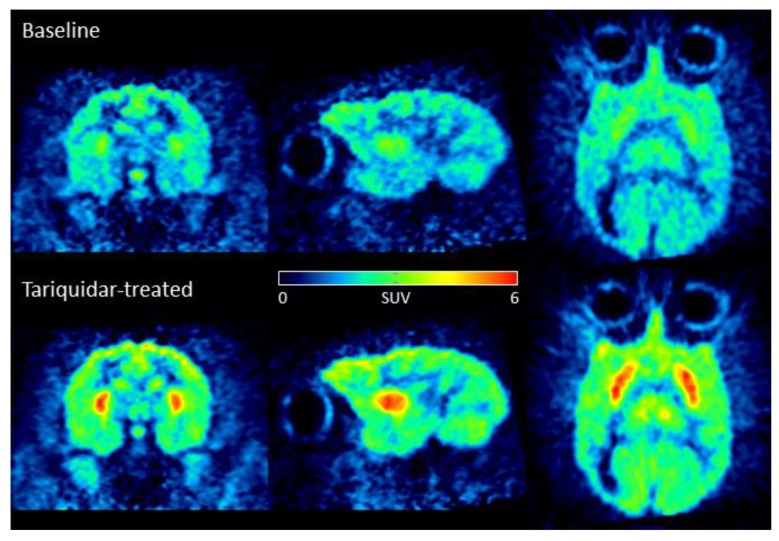
Summed coronal, sagittal, and transaxial PET images (0−90 min) of [^11^C]**1** in monkey under baseline and tariquidar (8 mg/kg, i.v.) treated conditions.

In the same monkey pretreated with tariquidar, peak brain uptake of [^11^C]**1** increased in all regions to between 3 and 4 SUV (Figure 4b). Areas-under the curve (AUC) between 0 and 90 min were higher than at baseline, ranging from ~ 20% higher in occipital cortex to 70% higher in putamen. Therefore, on the assumption that input of radioligand into brain was not increased appreciably under tariquidar treatment, [^11^C]**1** appears to be a substrate for P-gp in monkey, as in rodents. The ratio of radioactivity in putamen to cerebellum increased to 2.0 at 7 min and to 1.7 and 35 min, respectively, compared to about 1.4 at baseline. PET images (Figure 5) clearly showed high radioactivity uptake in striatum. However, radioactivity washout was faster in putamen than in other regions. The high radioactivity uptake in D_3_R and D_2_R poor cerebellum also indicates there is appreciable non-specific binding or off-target binding. Because of the clear efflux transporter substrate behavior of [^11^C]**1** across species, and the non-displaceable nature of the radioactivity in rat brain, we did not pursue further drug challenge experiments in monkey to elucidate the nature of the higher uptake of radioactivity in striatum than in cerebellum. Taken altogether the data show [^11^C]**1** is ineffective as a PET D_3_R in rodent and monkey.

The common features of D_2_R and D_3_R receptors add substantial challenges to the development of D_3_R-selective therapeutics and to the investigation of molecular mechanisms [17] involving D_3_Rs in the human brain [47,48]. Considering the *B*_max_ differences of D_2_R and D_3_R in monkey [21], rat, and human [49], a successful PET radioligand may require D_3_R over D_2_R selectivity of >500. Although this selectivity ratio has been achieved for a few compounds [48,50], to our knowledge, so far none of these molecules has been developed as a PET radioligand. Of note, Mach et al*.* also demonstrated the effect of endogenous dopamine on the uptake of the D_3_R preferring radioligand, [^18^F]FTP [25]. Pretreatment with lorazepam (1 mg/kg, i.v.) at 30 min before radioligand reduced endogenous dopamine activity before tracer injection and increased [^18^F]FTP uptake in the caudate, putamen, and thalamus. Hence, it may be necessary to consider reducing endogenous dopamine competition in PET studies especially with isoflurane-anesthetized animal subjects. In this regard, Sóvágó et al*.* used reserpine in an effort to reduce synaptic dopamine levels in their evaluation of [^11^C]RGH1756 in cynomolgus monkey [51], but this treatment resulted in no improvement of radioligand performance because D_3_R-specific binding was still not detected.

## 3. Materials and Methods

### 3.1. Synthesis of N-(4-(4-(3-Chloro-2-hydroxyphenyl)piperazin-1-yl)butyl)-1H-indole-2-carboxamide (***2***)

*N*-(4-(4-(3-Chloro-2-methoxyphenyl)piperazin-1-yl)butyl)-1*H*-indole-2-carboxamide (**1**) was prepared as previously described [30]. Compound **1** (220 mg, 0.5 mmol) was dissolved in chloroform and cooled to −78 °C. Boron tribromide (375 mg, 1.5 mmol) was added and the temperature was slowly allowed to increase to room temperature with continued stirring, for 8 h. The reaction mixture was quenched in ice-cold water, neutralized with ammonium hydroxide solution (10%) to pH 9.0 and extracted with chloroform (4 × 50 mL). The organic layers were combined, dried, concentrated and purified using flash chromatography (40% acetone/chloroform as eluent) to provide 138 mg (65%) of the product **2** as a white solid. m.p. 148–149 °C. ^1^H-NMR (400 MHz, CDCl_3_) *δ* 9.20 (s, 1H), 7.64 (d, *J* = 8.0 Hz, 1H), 7.44 (d, *J* = 7.7 Hz, 1H), 7.33–7.21 (m, 2H), 7.18–7.06 (m, 2H), 7.00 (d, *J* = 7.4 Hz, 1H), 6.79 (dd, *J* = 17.8, 9.6 Hz, 2H), 6.44 (s, 1H), 3.51 (d, *J* = 19.2 Hz, 4H), 2.92 (s, 3H), 2.63 (s, 3H), 2.49 (s, 2H), 1.70 (s, 4H). ^13^C-NMR (101 MHz, CDCl_3_) *δ* 161.55, 147.84, 139.99, 136.13, 130.88, 127.62, 126.68, 124.49, 121.77, 120.60, 119.99, 119.46, 119.04, 111.80, 101.65, 57.98, 53.67, 52.32, 39.68, 27.58, 24.29. HRMS (TOF MS EI+) calculated for C_23_H_28_ClN_4_O_2_ [M + H^+^] 427.1895, found 427.1897.

### 3.2. Binding Affinity of 1 to other Neuroreceptors

Off target screening data were obtained through the NIDA Addiction Treatment Discovery Program contract (ADA151001) with Oregon Health & Science University.

### 3.3. Radiosynthesis

No-carrier-added (NCA) [^11^C]iodomethane ([^11^C]CH_3_I) was produced from cyclotron-produced [^11^C]carbon dioxide via reduction to [^11^C]methane followed by vapor-phase iodination [52]. Thus, cyclotron-produced [^11^C]carbon dioxide (~65–87 GBq) was delivered to a PETtrace MeI process module (GE Medical Systems; Severna Park, MD) through stainless tubing (OD 1/8 in, ID 1/16 in) over 2 min, trapped on molecular sieve (13X), and reduced to [^11^C]methane over nickel at 360 °C. [^11^C]Methane was then recirculated over iodine at 720 °C to generate [^11^C]CH_3_I, which was trapped on Porapak Q held in the recirculation path. [^11^C]CH_3_I was swept with a stream of He gas (17 mL/min) into a stainless-steel loop [37] pre-loaded with a mixture of precursor **2** (1.5–1.9 μmol) and *n*Bu_4_NOH (1 M in MeOH, 2.1–3.0 µmol) dissolved in DMF (80 μL). When radioactivity reached a maximum in the loop (determined by a proximal radiation detector) the He gas flow was stopped and the reaction allowed to proceed for 5 min at room temperature. The contents of the loop were then injected onto a semi-preparative HPLC column (Luna C18, 10 × 250 mm, 10 μm; Phenomenex, Torrance CA) eluted with MeCN:25 mM aqueous HCO_2_NH_4_ (52:48, *v*/*v*) at 6 mL/min, with eluate monitored for radioactivity and absorbance at 254 nm. The radioactive fraction (*t*_R_ = 10–12 min) corresponding to the desired product was collected in a flask, evaporated to dryness in vacuo, redissolved in 10% ethanol/saline (10 mL, *v*/*v*), and passed through a sterile 0.22 μm filter (Millex-MP; Millipore, Burlington, MA, USA) into a sterile vial. For rodent PET studies, the procedure was performed without terminal filtration. Radiochemical and chemical purities were measured with analytical HPLC on a Luna C18 column, (4.6 × 250 mm, 10 μm; Phenomenex) eluted with MeCN:25 mM aqueous HCO_2_NH_4_ (60:40, *v*/*v*) at 2 mL/min with eluate monitored for radioactivity and absorbance at 254 nm. The identity of [^11^C]**1** was confirmed by observing HPLC co-mobility with **1**, and with LC-MS analysis of the associated carrier.

### 3.4. Lipophilicity (logD_7.4_) Measurements and Stability in Aqueous Buffer

The value for the distribution coefficient (log*D*_7.4_) of [^11^C]**1** between 1-octanol and sodium phosphate buffer (0.15 M, pH 7.4) was determined with a technique described previously [53]. The radioligand in ethanol/saline was placed in sodium phosphate buffer (0.15 M, pH 7.4) for the duration of the experiment followed by the determination of its radiochemical purity to obtain information on its stability in buffer. [^11^C]**1** (293 MBq in 600 μL) was added to sodium phosphate buffer (0.15 M, pH 7.4; 7.0 mL) and mixed well. Aliquots (1.0 mL) were distributed to eight borosilicate disposable culture tubes (13 × 100 mm). Six tubes each contained 1-octanol (1.0 mL), and two tubes (without octanol) served for determining the stability of [^11^C]**1** and adsorption to the walls of the tube by incubation for the duration of the experiment. Radioactivities from six tubes were extracted with 1-octanol. Extraction was performed by vortexing the tubes with their content for 1 min. The tubes were centrifuged for 1 min. The organic and the aqueous phases were separated. Each phase was then sampled 50 μL and 200 μL respectively and counted separately in a well-type γ-counter (model 1480 Wizard; Perkin-Elmer) with an electronic window set between 360 and 1800 keV. The remaining two tubes, contained the radioactivity in buffer and served as stability measures for the radioligand. Radioactivity in the aqueous phases (200 μL) resulted in counting error of 0.3 ± 0.03% (*n* = 6) at one standard deviation. The radiochemical composition of the aqueous buffer after extraction with 1-octanol was determined with HPLC on an X-Terra C18 column (10 μm, 7.8 × 300 mm; Waters Corp., Milford, MA, USA) eluted with MeOH:H_2_O:Et_3_N (80:20:0.1 *v*/*v/v*) at 4.0 mL/min. The HPLC system consisted of Beckman Gold (Beckman Coulter, Inc.; Fullerton, CA, USA) analytic pumps equipped with an in-line photodiode-array detector and a flow-through NaI scintillation detector-rate meter (Bioscan, Washington, DC, USA). The resulting radiochromatograms provided correction factors for the γ-counter counts to determine parent radioactivity only (corrected radioactivity). Data from the radioanalyses were collected and stored with Bio-Chrome Lite software (Bioscan) and analyzed after decay correction of the radiochromatograms. Log*D*_7.4_ was calculated according to the following formula:

(1) $$\;\log D_{7.4}=\log\left(\frac{\mathrm{cpm}\;\mathrm{organic}\;\mathrm{phase}}{\mathrm{corrected}\;\mathrm{cpm}\;\mathrm{aqueous}\;\mathrm{phase}}\right)\;$$

### 3.5. Tissue Stability

The stability of [^11^C]**1** was evaluated in rat whole blood, human and rat plasmas, and rat, monkey and human brain homogenates. These tissues were stored frozen at −70 °C but thawed on the day of analysis. [^11^C]**1** (~ 370 kBq/10.0 μL) was added to thawed tissues (500 μL), mixed well and incubated at 37 °C for 30 min. Plasma analysis was performed as reported previously [54]. The stability of [^11^C]**1** was expressed as the tissue radiochromatographic composition divided by the radiochemical purity of the radioligand.

### 3.6. Plasma Free Fraction

The plasma free fraction (*f*_p_) of [^11^C]**1** was measured with ultrafiltration through membrane filters (Centrifree; Millipore; Burlington MA), as previously described [55]. Briefly, [^11^C]**1** (925 kBq, ~23 μL) was added to the plasma (1000 μL). The mixture was incubated at room temperature for 10 min and then processed as described previously. The ultrafiltration components that contained high radioactivity were allowed to decay to within the optimal range of the γ-counter before they were counted. Quantification of the ultrafiltrates was performed gravimetrically.

### 3.7. Cell Uptake Percentage

Blood hematocrit (Hct) was determined using heparinized capillary tubes (12 × 40 mm) to sample blood drawn after the injection of the vehicle, and immediately before injection of [^11^C]**1**. The capillary tubes were then centrifuged, and Hct determined using a StatSpin^®^ CritSpin™ Microhematocrit system (Los Angeles, CA, USA). Aliquots of anticoagulated rat blood (250 µL) were placed in three Eppendorf tubes (1.5 mL). [^11^C]**1** (18.5 kBq) was added to each tube and incubated at 37 °C for 30 min. Then whole blood (50 µL) was removed from each tube and counted in the γ-counter. The tubes were then centrifuged before removing plasma (50 µL) for γ-counting. The relative cellular blood (RBC, WBC and platelets) partitioning of [^11^C]**1** was then calculated as:

(2) $$\%\mathrm{Cell}=\frac{C_{\mathrm{whole}\;\mathrm{blood}}-C_{\mathrm{plasma}}\times \left(1-\mathrm{Hct}\right)}{C_{\mathrm{whole}\;\mathrm{blood}}}\;$$

where *C*_whole blood_ is the concentration of radioactivity in whole blood (cpm/mL), *C*_plasma_ is the concentration of radioactivity in plasma (cpm/mL), and *Hct* is the hematocrit.

### 3.8. Rat Ex Vivo Measurements

[^11^C]**1**, in 10% ethanol-saline solution, was injected intravenously through the penile vein in two anesthetized male Sprague-Dawley rats. One rat served as baseline and the other was pre-treated at about 10 min before [^11^C]**1** injection with tariquidar (16 mg/kg; 2.1 mL). Rats were anesthetized with 1.5% isoflurane in oxygen. Thirty minutes after injection of [^11^C]**1** (~74 MBq) a large anticoagulated (heparin) blood sample was drawn. The rats were then immediately sacrificed by decapitation, and their cortices, cerebella, and striata were excised. Each of the brain tissues was placed in MeCN (1 mL) and counted in a γ-counter. Each brain tissue along with added **1** (50 μg) was then homogenized with a hand-held tissue Tearor (model 985-370; BioSpec Products Inc., Bartlesville OK). Water (500 μL) was then added and the tissues were further homogenized and counted in the γ-counter for calculating recoveries into MeCN. The homogenates were centrifuged at 10,000× *g* for 1 min. The clear supernatant liquids were injected onto a C18 column (X-terra, 10 μm, 7.8 × 300 mm; Waters Corp., Milford, MA, USA ) eluted with MeOH:H_2_O:Et_3_N (78.5:21.5:0.1 *v*/*v*/*v*) at 4.0 mL/min. After separating plasma from blood cells, plasma samples (200 μL) were counted in γ-counter and plasma aliquots (450 μL) were each placed in MeCN (720 μL) along with of carrier **1** (5 μg) for deproteinization. After mixing the samples well, H_2_O (100 μL) was added. The samples were further mixed well, counted in a γ-counter, and centrifuged at 10,000× *g* for 1 min. Supernatants were injected onto the radio-HPLC and analyzed under the above conditions. All precipitates were counted in the γ-counter for calculating recoveries in the supernatants that were injected onto the HPLC.

An untreated rat served as a control for in vitro experiments. Anticoagulated blood, plasma, and frontal brain were harvested. The brain (1.6 g) was homogenized in cold saline. After adding [^11^C]**1** to the whole blood, plasma and brain homogenate, the tissues were incubated at 37 °C for 1.0 h. In vitro whole blood (200 μL) was placed in water (300 μL) to lyse open all cells. Then an aliquot (450 μL) was removed and placed in MeCN (720 μL). Tissue samples were then processed as detailed above and subjected to radio-HPLC analysis.

### 3.9. PET Imaging

PET imaging experiments were performed in accordance with the Guide for Care and Use of Laboratory Animals [56] and were approved by the National Institute of Mental Health Animal Care and Use Committee. All animals were anesthetized with 1.5% isoflurane. Rat (Sprague-Dawley, male; 346 ± 21 g) scans were acquired with a microPET Focus 220 scanner (Siemens Medical Solutions, Knoxville, TN, USA) for a duration of 90 min. [^11^C]**1** (~23.8 ± 0.9 MBq) was injected intravenously (i.v.) through a tail vein catheter. In some rats, tariquidar (16 mg/kg, i.v.) was administered at 15 min before radioligand injection. In some other rats, drug challenge agents (BP897, 0.5 mg/kg, i.v., or spiperone, 1.0 mg/kg, i.v.) were administered at 10 min before radioligand injection for preblocking experiments or 15 min after radioligand injection for displacement experiments. For experiments in mice (wild type and P-gp/BCRP knock-out; male; Taconic FVB.129P2-*Abcb1a^tm1Bor^Abcb1b^tm1Bor^Abcg2^tm1Ahs^*N7; 27 ± 1.1 g), scans were acquired with a microPET Focus 120 camera for a duration of 90 min. [^11^C]**1** (~9.9 ± 1.5 MBq) was injected through a tail vein catheter. Monkey (male rhesus macaque, 9.9 kg) experiments were acquired using microPET Focus 220 (Siemens Medical Solutions, Knoxville TN) for a duration of 90 min. A bolus [^11^C]**1** (~229 MBq) was injected intravenously at baseline. In the same monkey, a second PET experiment was performed 3 h later, in which tariquidar (8 mg/kg, i.v.) was administered at 10 min before injection of [^11^C]**1** (~329 MBq).

Images were reconstructed using Fourier rebinning followed by 2D OSEM with attenuation correction for rats and monkey and without attenuation correction for mice. Scatter correction was applied only for monkey. Volume of interests were delineated manually for rats and mice, and with a co-registered MRI template for monkey. All radioactivity was decay-corrected and expressed as standardized uptake value (SUV) which is radioactivity concentration corrected for subject weight and administered dose. Time activity curves in SUV were obtained and compared between conditions. The PET data analysis was performed using PMOD.

## 4. Conclusions

[^11^C]**1** was labeled by ^11^C-methylation of its desmethyl precursor (**2**) in high yield and molar activity. In vivo evaluation with PET showed that [^11^C]**1** was an avid substrate of efflux transporters and lacked D_3_R-specific signal in rodents and monkey. Binding to off-target D_2_R appeared absent in rat striatum, thereby indicating that D_2_R affinity (*K*_i_ = 53 nM) is adequately low. Candidate PET radioligands with absence of efflux transporter behavior and with even higher D_3_R affinity are likely required for successful PET imaging of D_3_R.

## Figures and Tables

**Table 1:** Unmetabolized [^11^C]**1** in rat plasma and brain tissues at 30 min after i.v. injection.

Tissue	Unmetabolized [^11^C]1 (%)	
	Baseline (*n* = 1)	Tariquidar-Treated (*n* = 1)
Plasma	76.1	68.9
Cortex	99.0	98.7
Cerebellum	98.8	98.8
Striatum	99.3	98.8

**Table 2:** Rat brain tissue concentrations (SUV) of [^11^C]**1** under baseline and tariquidar-treated conditions measured ex vivo 30 min after intravenous injection of radioligand.

Tissue	[^11^C]1 Concentration (SUV ^1^)	
	Baseline (*n* = 1)	Tariquidar-Treated (*n* = 1)
Plasma	0.06	0.04
Whole Blood	0.09	0.07
Cerebellum	1.20	2.84
Cortex	1.33	3.49
Striatum	1.36	3.53

^1^ SUV = standardized uptake value.
